# Antimicrobial Activity of Polyphenols and Natural Polyphenolic Extracts on Clinical Isolates

**DOI:** 10.3390/antibiotics11010046

**Published:** 2021-12-30

**Authors:** Tamara Manso, Marta Lores, Trinidad de Miguel

**Affiliations:** 1 Hospital Público da Mariña, E-27880 Burela, Spain; tamara.manso.gomez@sergas.es; 2 Department of Microbiology and Parasitology, Universidade de Santiago de Compostela, E-15782 Santiago de Compostela, Spain; 3 Laboratory of Research and Development of Analytical Solutions (LIDSA), Department of Analytical Chemistry, Nutrition and Food Science, Universidade de Santiago de Compostela, E-15782 Santiago de Compostela, Spain; marta.lores@usc.es

**Keywords:** antimicrobial activity, polyphenols, plant extracts, clinical isolates, antibiotics

## Abstract

Antibiotic resistance is a growing global problem that affects people, animals, the environment, and the economy. Many clinically relevant bacteria have become resistant to antibiotics, and this fact is emerging as one of the major threats to public health. The lack of new antibiotics, which is due to their time-consuming and costly development, exacerbates the problem. Therefore, it is necessary to identify new antimicrobial agents to treat bacterial and fungal infections. Plant extracts, which are valuable sources of bioactive compounds, mainly polyphenols, play an important role as a new strategy to combat pathogenic microorganisms. There is an extensive body of supporting evidence for the potent antibacterial and antifungal activities of polyphenols. Furthermore, some polyphenols show a synergistic effect when combined with antibiotics and antifungals, suggesting a promising alternative for therapeutic strategies against antibiotic resistance. However, only a few articles are found when searching the antibacterial or antifungal activities of polyphenols employing clinical isolates. Hence, this review focuses on the antimicrobial activity of polyphenols and extracts rich in polyphenols on clinical isolates, organized according to the World Health Organization priority pathogens classification.

## 1. Introduction

Antibiotics are used to prevent and treat infections caused by bacteria. Their discovery has been a historic milestone which has revolutionized medical practice, industrial microbiology, and human life in general. Nevertheless, from the time of their first uses, resistant bacterial strains have been appearing. This trend has intensified during recent decades due to the overuse and misemployment of these essential drugs. In clinical practice, patients urging their doctors for antibiotics in order to treat viral infections or not completing the antibiotic regimen are seen every day. The misuse of antibiotics in farmed animals during recent decades is also worrying. All these factors can lead to antibiotic resistance, which is one of the greatest threats involving health or food industries. A disastrous consequence of antibiotic resistance is the difficulty of treating bacterial infections since antibiotics lose their efficacy. Furthermore, antibiotic resistance leads to more extended hospital stays, increases medical costs, and endangers certain medical achievements, such as transplants or surgeries. It is essential to change the current behavior around antibiotics regarding not only their prescription and use but also improving the prevention and the control of infections. Antibiotic resistance is a global problem that affects people, animals, the environment and the economy. According to the World Health Organization (WHO), if preventive measures are not adopted urgently, deaths related to resistant bacteria will be the leading cause of death on the planet by 2050. Different institutions are taking conscious steps regarding this topic. The United Nations Organisation, in its 2016 Assembly Declaration, proclaimed that the development of antibiotic resistance and the lack of new antibiotics is a huge public health problem and requires global involvement and coordination [1].

The European Antimicrobial Resistance Surveillance Network (EARS-Net) is the most important public system for antimicrobial resistance (AMR) surveillance in Europe. Maps, graphs, and tables are available in their public website. Among their objectives, the most important are: (i) the collection of AMR data, (ii) the analysis of temporal and spatial trends of AMR in Europe and (iii) the urge to implement, maintain and improve national AMR surveillance programs.

In a great number of European countries, national plans are in progress. In Spain, the National Antibiotic Resistance Plan (PRAN) has the objective of reducing the growth of antibiotic resistance and its impact on the health of the entire population by controlling antibiotic consumption in human and veterinary medicine [2]. Écoantibio 2 is the second French national plan designed to combat the antimicrobial resistance in veterinary medicine from 2017 to 2022 [3]. In Germany, the German Antimicrobial Resistance Strategy (DART) [4] was created to fight against antibiotic resistance. The Swiss Antibiotic Resistance Strategy (StarR) follows the same objective [5].

**Figure 1:**
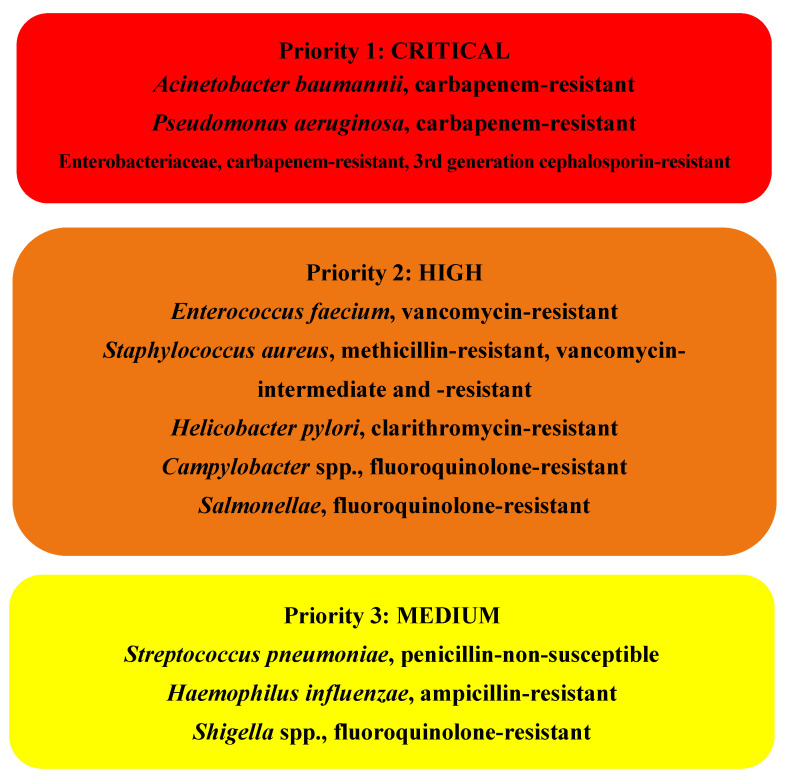
WHO list of priority pathogens for research and development of new antibiotics [6,7].

In 2017, the WHO published a list of global priority pathogens containing 12 families of bacteria which are the most threatening to human health. This list focuses mainly on Gram-negative bacteria resistant to multiple antibiotics, as they are able to find new ways to overcome antibiotics by passing along genetic material, allowing other bacteria to also become drug resistant. This list is divided into three categories depending on the urgency of need for new antibiotics: critical, high, and medium priority (Figure 1) [6,7].

The critical group, the most important one, includes multidrug resistant bacteria, which show particular danger in hospitals, nursing homes, and among patients. *Acinetobacter*, *Pseudomonas* and various *Enterobacteriaceae* (including *Klebsiella*, *Escherichia coli*, *Serratia* and *Proteus*) are included in this category. Important medical conditions such as bloodstream infections and pneumonia can be caused by these bacteria. They can develop resistance against a broad spectrum of antibiotics such as carbapenems or third generation cephalosporins [6,7].

The other groups—the high and medium priority categories—are composed of other increasingly drug-resistant bacteria that cause more common diseases, such as gonorrhea, and food poisoning, such as *Salmonella* [6,7].

Another term related to multidrug resistant bacteria is “ESKAPE”, a bacterial group which includes 6 pathogens with growing multidrug resistance and virulence: *Enterococcus faecium*, *Staphylococcus aureus*, *Klebsiella pneumoniae*, *Acinetobacter* *baumannii*, *Pseudomonas aeruginosa* and *Enterobacter* spp. [8]. These pathogens cause most nosocomial infections and are able to “escape” the biocidal action of antimicrobial agents [9].

Since the development of new antibiotics is not cost effective, there is a lack of new drugs, which also exacerbates the problem. Therefore, it is of utmost importance to find new strategies to treat bacterial infections. Alternative strategies to combat resistance to these drugs have been studied: bacteriophages, antimicrobial peptides, photodynamic light, and silver nanoparticles [6].

Plant extracts also play an important role as an emerging strategy to fight pathogenic microorganisms. The medicinal properties of plants have been known since ancient times when they were widely used to treat multiple pathologies. Today, many plant extracts still show a wide variety of benefits for humans. In this context, polyphenols are compounds derived from different parts of plants which contain one or more phenolic groups. They are divided into several groups according to their structure (Table 1). They have not only a multitude of human health benefits (including antioxidant, anti-inflammatory, antidiabetic, antiallergic, antiatherogenic, antihypertensive, antithrombotic, anti-cancer, cardioprotective, osteoprotective, neuroprotective, anti-aging and hepatoprotective benefits), but antibacterial, antitoxin, antiviral and antifungal properties have also been described [10,11]. Polyphenols are also used as natural preservatives in the food industry due to their antioxidant and antimicrobial properties [12,13,14,15,16].

Although much work has been done demonstrating the antimicrobial activity of polyphenols, research works employing clinical isolates are lacking. Such clinical isolates refer to samples of infections collected directly from patients in healthcare facilities. Several studies have reported that plant extracts rich in polyphenols can inhibit the growth of pathogenic bacteria and fungi, suggesting that they may be clinically useful by acting as natural alternatives to synthetic antimicrobials [17,18,19].

The aim of this review is to gather all the information related to the antibacterial and antifungal activity of polyphenols and plant extracts rich in these molecules on clinical isolates. This review does not cover polyphenol activity against reference strains, or any microorganism not collected from patients’ samples. Therefore, when a study describes both types of microorganisms, we are going to focus only on the clinical isolates. All free articles meeting the criteria are summarized in the following sections.

## 2. Antibacterial Activity of Polyphenol Extracts in Clinical Isolates

Polyphenols show antibacterial activity against a large number of bacteria (including Gram-positive and Gram-negative bacteria) and fungi. A wide variety of classifications have been published of the more than 10,000 compounds that can be considered. Polyphenols, whose common structural attribute is that they have at least one aromatic ring with one or more hydroxyl groups, can be grouped, for example, into families according to the number and arrangement of carbon atoms in their structure, giving rise to 10 major classes of polyphenols. Some of these compounds are derived from the oligo/polymerization of simple phenols. In addition, many of these compounds form glycoconjugates with mono- or polysaccharides and generate functional derivatives such as esters and methyl esters. Therefore, the structural and, consequently, functional diversity of polyphenolic compounds is enormous. Table 1 summarizes the polyphenols with demonstrated antibacterial and antifungal activity against clinical isolates considered in this review and their corresponding structural groups. Most of the active polyphenols are flavonoids or hydrolysable tannins. Hydrolysable tannins can be divided into gallotannins and ellagitannins, with the latter being the most abundant in the extracts whose antimicrobial potential has been tested against clinical samples. The list does not, however, include all the botanical extracts reviewed, as a number of papers do not detail the individual polyphenolic composition of the extracts tested but only refer to the presence of families such as flavonoids or tannins in a general way.

Indeed, a multitude of studies have been published on polyphenolic antimicrobial activity, but very few of them report information regarding clinical isolates (Table 2 and Table 3), with *Staphylococcus aureus* being the most studied microorganism. The methods used for the evaluation of the antibacterial or antifungal activity are microdilution or disk diffusion tests, determining minimum inhibitory concentration (MIC), minimum fungicidal concentration (MFC) and minimum bactericidal concentration (MBC), depending on the study.

We are going to describe the information found taking into consideration the WHO classification; therefore, the first mentioned pathogens will be those belonging to the most critical group (priority 1).

### 2.1. Bacteria Belonging to Priority 1: Critical

#### 2.1.1. *Acinetobacter baumannii*

In the fight against *A. baumannii*, it was demonstrated that norwogonin [20] and theaflavin [21] (better when combining theaflavin: epicatechin (2:1)) possessed activity against clinical isolates of this multiresistant bacteria. Crude extracts of *Polygonum cuspidatum* [22] and leaves and inflorescences of different species of *Amaranthus retroflexus* vegetative organs [23] were also active against it.

Y. Miyasaki et al. identified three extracts (*Scutellaria baicalensis*, *Rosa rugosa* and *Terminalia chebula*) on the basis of mass spectrometry (MS) and ultraviolet (UV) data, as these were the extracts which showed ≥40% of growth inhibition against the bacteria tested. The most potent compound was identified as norwogonin, with a MIC of 128 mg/mL and MBC of 256 mg/mL against clinically relevant strains of *A. baumannii.* The three extracts mentioned contained norwogonin (*S. baicalensis)*, ellagic acid (*R. rugosa*) and a mixture of chebulagic acid, chebulinic acid, corilagin and terchebulin (*T. chebula*). In this study, norwogonin was purified from *S. baicalensis* extract in dimethyl sulphoxide (DMSO). Ellagic acid was purified from *R. rugosa* extract in warm water, and chebulagic acid, chebulinic acid, corilagin and terchebulin were purified from *T. chebula* extract also in warm water. These solutions were studied against three clinical isolates of multi-drug resistant (MDR) *A. baumannii* from blood and respiratory samples, and the authors found that norwogonin was the most active polyphenol of all tested; however, it showed no synergy when combined with several antibiotics. This is the first report of antibacterial activity of norwogonin against *A. baumannii* [20]. Other authors have demonstrated that theaflavin possessed antibacterial activity against *A. baumannii* from sputum samples, which was increased when it was combined with epicatechin (theaflavin:epicatechin (2:1)). According to this report, theaflavin possesses a strong antibacterial activity that could be relevant in the clinic against resistant microorganisms. This seems to be the first evidence of the antibacterial properties of theaflavin against clinical isolates of *A. baumannii* and *S. maltophilia*. Furthermore, it is the first report of antibacterial synergy between theaflavin and epicatechin against these important nosocomial microorganisms [21].

An experiment using crude ethanol extracts of *P. cuspidatum* and successive partitions with *n*-hexane, chloroform, ethyl ether (EE) and ethyl acetate (EA) based on solvent polarity demonstrated that the ethanol extract possessed antibacterial activity against *A. baumannii*. The strains used in this report were isolated from blood and sputum samples. Considering all partitioned fractions, EE and EA fractions showed greater antibacterial properties against all tested strains. This suggested that the relatively medium polarity constituents have more antibacterial potential than the very polar or nonpolar constituents of *P. cuspidatum.* The extract that revealed the most significant antibacterial activity against the tested strains was EE. Even if the composition of the extract is not defined in this study, according to the literature reports, P. Su et al. presume that the active substances that contribute to the antibacterial activities could be resveratrol and piceid, as well as other polyphenolic compounds. In this study, the authors also tried to determine if there was any synergy with antibiotics, but no positive effect was observed [22]. Extracts obtained from leaves and inflorescences of different species of *A. retroflexus* vegetative organs also showed antibacterial properties against *A. baumannii.* Like the previous study, this research failed to demonstrate any synergy of the polyphenolic extracts when used in combination with antibiotics against *A. baumannii.* In this study, the quantitative determination of total polyphenols of the extract is expressed as gallic acid equivalents/mL. It is also remarkable that the highest content of polyphenols was obtained for the extract of *A. retroflexus* inflorescences. This was also the most active extract against the tested microbial strains, suggesting that antibacterial activity and the content of polyphenols are related, as described in previous studies [23].

#### 2.1.2. *Pseudomonas aeruginosa*

*P. aeruginosa* is another common pathogen in clinical settings with an urgent need for new antibiotics. Several studies have demonstrated that polyphenols and polyphenolic extracts can fight this bacterium. Epigallocatechin-gallate (EGCG) [24], extracts of *Curcuma longa* [25], ethanol crude extracts of *P. cuspidatum* [22], and extracts obtained from leaves and inflorescences of the vegetative organs of different species of *A. retroflexus* [23] were found to be active against clinical strains of *P. aeruginosa.*

When combining an alcoholic extract of *A. retroflexus* leaves with trimetoprim-sulphamethoxazole, piperacillin-tazobactam, ciprofloxacin, ticarcillin-clavulanic acid, piperacillin, ceftriaxone and colistin against clinical strains of *P. aeruginosa*, the antibacterial activity was potentiated. The extract from the inflorescences of *A. retroflexus* improved the activity of ceftriaxone, ticarcillin-clavulanic acid, colistin, piperacillin, norfloxacin and oxacillin against the bacteria [23].

J. W. Betts et al. studied 15 clinical MDR strains of *P. aeruginosa.* These strains, which are resistant to aztreonam, were susceptible to a combination of aztreonam with EGCG, demonstrating the synergy between the antibiotic and this polyphenol, not only in vitro but also in vivo. EGCG can also return the antibacterial activity of aztreonam to concentrations below the European Committee on Antimicrobial Susceptibility Testing (EUCAST) sensitivity breakpoint for *P. aeruginosa.* Synergy was also found between EGCG and the third-generation cephalosporin, cefotaxime [24]. *C. longa* L (Zingiberaceae), which contains flavonoids and tannins, also showed antibacterial activity against clinical strains of *P. aeruginosa*. The authors compared the ethanolic extract and the essential oil of this plant species and concluded that, even if both had antibacterial properties, the ethanolic extract worked better against the bacteria evaluated. Additionally, they discovered a correlation between the concentration of the ethanolic extract and the essential oil: when the concentration of the extract increased, the absorbances were also higher, showing a reduction in bacterial growth [25]. As previously described for *A. baumannii*, ethanol crude extracts of *P. cuspidatum* [22] and ethanolic extracts obtained from leaves and inflorescences of the vegetative organs of different species of *A. retroflexus* [23] were also active against *P. aeruginosa*.

#### 2.1.3. *Enterobacteriaceae*

The third and last group of pathogens classified as critical/priority 1 by the WHO is Enterobacteriaceae. We have also found literature demonstrating that some polyphenols and extracts rich in these molecules possess antibacterial activity against clinical strains of this group of Gram-negative bacteria.

Due to the scarce amount of information about *Salmonella* and *Shigella* (only one study was found), we have decided to mention these bacteria here, for they belong to the Enterobacteriaceae although the WHO classifies them into lower priority categories.

Below we provide a list of enterobacteria and the extracts and polyphenols they are sensitive to, according to the literature:

- *K. pneumoniae:* extracts of *Acacia nilotica*, *Cinnamum zeylanicum* and *Syzygium aromaticum* [26], tea polyphenols (catechins, EGCG, ECG (epigallocatechin) GCG (epicatechin gallate), EC (epicatechin) [27], extracts of *C. longa* [25], extracts of *Prosopis laevigata* and *Opuntia* *ficus-indica* [28], extracts of *Lawsonia inermis (Klebsiella* spp.) [29] and extracts obtained from leaves and inflorescences of different species of *A. retroflexus* vegetative organs [23];
- *E. coli*: extracts of *A. nilotica*, *C. zeylanicum* and *S. aromaticum* [26], extracts of *C. longa* [25], extracts of *P. laevigata*, *O. ficus-indica* and *Gutierrezia microcephala* [28], extracts of *L. inermis* [29], extracts of *Punica granatum* [30] and extracts obtained from leaves and inflorescences of different species of *A. retroflexus* vegetative organs [23];
- *Proteus* sp.: extracts of *C. longa* [25];
- *Salmonella* sp.: extracts of *C. longa* [25] and extracts of *L. inermis* (*S. typhi*) [29];
- *Shigella sonnei*: extracts of *L. inermis* [29].

R. Khan et al. tested extracts from three different plants and found that the strongest antibacterial activity was observed with *A. nilotica*, followed by *C. zeylanicum* and *S. aromaticum*, respectively. Although the composition of the extract was not defined, the antibacterial properties of the extracts are believed to be mostly due to tannins, flavonoids, and other phenolic compounds, according to the literature [26].

Tea polyphenols (*Camellia sinensis*) such as catechins, epigallocatechin gallate, epigallocatechin and epicatechin showed antibacterial activity against 20 strains of *K. pneumoniae* collected from sputum, venous blood, urine, and feces samples. N. Zhang et al. also tested several antibiotics (imipenem, piperacillin, piperacillin/tazobactam, cefepime, cefotaxime and ceftazidime) combined with the mentioned polyphenols and demonstrated synergistic effects of the combinations, being able to overcome the antibiotic resistance of the strains. Piperacillin/tazobactam and cefepime had the greatest synergistic effect, followed by cefotaxime and ceftazidime [27].

In another study, out of eight methanolic plant extracts (*Sophora secundiflora*, *Sphaeralcea* *ambigua*, *P. laevigata*, *O. ficus-indica*, *Marrubium vulgare*, *Scutellaria drummondii*, *Nothoscordum bivalve* and *G. microcephala*) which contain polyphenols such as tannins and flavonoids, three (*P. laevigata*, *O. ficus-indica* and *G. microcephala*) were selected for phytochemical screening. The extract that showed the best activity against the tested enterobacteria was *P. laevigata*. However, *N. bivalve* bulb did not show activity against any microorganism tested [28].

*L. inermis* extract showed antibacterial activity against all bacteria tested. Four types of *L. inermis* extracts were used employing methanol, water, chloroform, and acetone as extraction solvents and DMSO and water as dissolution solvents. DMSO was used to dissolve the residues of methanol, acetone, and chloroform extracts, while aqueous extract residues were dissolved in distilled water at different concentrations. In this report, chloroform extract showed the best antibacterial activity against all the tested bacteria except for *E. coli* and *Bacillus subtilis.* For these bacteria, the best results were observed with acetone extract. All extracts contained tannins and other polyphenols and exhibited antibacterial activity against the total of the isolates tested. However, sensitivity was different for each extract [29].

The antibacterial activity of pomegranate fruits (*P. granatum* L.) was evaluated in 50% (*v*/*v*) aqueous ethanol solution, showing positive results against the two bacteria tested: a strain of *S. aureus* from the pharyngeal tract and another strain of *E. coli* from a sputum sample. It was also proved that the antibacterial activity of the crude extracts was greater than that of purified ones. The polyphenols detected in pomegranate juice were anthocyanins (especially delphinidin and cyanidin glucosides), hydrolysable tannins (gallotannins), ellagitannins, gallagyl esters, hydroxybenzoic and hydroxycinnamic acids, whereas the peel extract presented a composition rich in hydrolysable ellagitannins (HHDP-gallagyl-hex isomers (a-and b-punicalagin)), glycosylated derivates of ellagic acid (hexose, pentose and deoxyhexose derivates) and free ellagic acid. The extracts studied derived from crude and purified juice and from crude and purified peel, and contained different amounts of total polyphenols, with the peel extracts having higher polyphenolic concentrations [30].

The above-mentioned study by I. C. Marinas et al. also involves Enterobacteriaceae. Among the bacteria tested, *K. pneumoniae* was (together with *B. subtilis*) the microorganism which exhibited the best results. Regarding the synergy between polyphenols and antibiotics, the alcoholic extract of *A. retroflexus* leaves increased the antibacterial activity of trimetoprim-sulphamethoxazole, piperacillin-tazobactam, ciprofloxacin, cefotaxime, cephalexin against *K. pneumoniae* and trimetoprim-sulphamethoxazole and piperacillin-tazobactam against *E. coli.* The extract from the inflorescences of *A. retroflexus* potentiated the activity of penicillin, fosfomicin, erithromycin, amoxicillin, ofloxacin and aztreonam against *K.* *pneumoniae* [23].

### 2.2. Bacteria Belonging to Priority 2: High

Next category includes the bacteria with high priority. As previously said, *S. aureus* is the most-studied microorganism in terms of the antibacterial activity of polyphenols and polyphenolic extracts. Regarding other bacteria included in this category, only one study was found, dealing with *Helicobacter pylori*.

#### 2.2.1. *Staphylococcus aureus*

A high number of investigations demonstrated the antibacterial properties of different polyphenols and extracts rich in these molecules against clinical isolates of *S*. *aureus*.

According to the literature, *S. secundiflora*, *S. ambigua*, *P. laevigata*, *O. ficus-indica*, *M. vulgare*, *S. drummondii* and *G. microcephala* extracts [28], ethanol crude extracts of *P. cuspidatum* [22], *L. inermis* extracts [29], pomegranate fruits (*P. granatum* L.) extracts [30], extracts obtained from leaves and inflorescences of different species of *A. retroflexus* vegetative organs [23] and *Tabernaemontana alternifolia* (Roxb) stem bark aqueous extracts [31] were found to be active against clinical isolates of *S. aureus*. Morin, quercetin, kaempferol, (−)-epigallocatechin gallate, (+)-catechin acyl derivates, epicatechin gallate, 3-*O*-decyl-(+)-catechin, (+)-catechin, protocatechuic acid ethyl ester and caffeic acid [32], and punicalagin are individual polyphenols that showed the same behavior. Additionally, *Cistus salviifolius* and *P. granatum* extracts [33], alcoholic extract from Thai mango (*Mangifera indica* L. cv. ‘Fahlun’) seed kernel extract (MSKE), pentagallolylglucopyranose, methyl gallate and gallic acid [34], raw shelled (NP) and roasted salted (RP) pistachios [35,36], *Dendrobenthamia capitata* extracts and betulinic acid [37] also showed antibacterial activity against clinical isolates of *S. aureus*.

A study by E. Sánchez et al., already mentioned above, demonstrated antibacterial activity of seven methanolic plant extracts against *S. aureus* [28].

*S. aureus* was also the bacterial species which exhibited higher sensitivity to *P. cuspidatum* crude extract out of all tested bacterial isolates [22].

Stem bark (*T. alternifolia* (Roxb)) aqueous extracts, which contain flavonoids, were evaluated against 12 strains of *S. aureus* (11 methicillin-resistant *S. aureus* (MRSA), 4 vancomycin-resistant *S. aureus* (VRSA)) isolated from skin infection samples. The authors prepared direct aqueous extracts and sequential aqueous extracts of the stem bark of *T. alternifolia*, which showed antibacterial activity against the clinical isolates. However, when they used petroleum ether and ethyl acetate as other solvents, the resulting extracts were not active against the aforementioned strains [31].

From a review summarizing different polyphenols active against *S. aureus* strains, the ones active against clinical isolates were flavonoids: specifically, they included flavonols, such as morin, quercetin, kaempferol; flavanols and derivatives such as (−)-epigallocatechin gallate, (+)-catechin acyl derivates, epicatechin gallate, 3-*O*-decyl-(+)-catechin, (+)-catechin; and phenolic acids and derivatives, such as protocatechuic acid ethyl ester and caffeic acid [32].

Álvarez-Martínez F. J. et al. determined the antibacterial activity of several botanical extracts, such as *P. granatum*, *C. salviifolius* and *Citrus paradisi* and some selected pure polyphenolic compounds (GA: gallic acid, P: punicalagin, Q3G: quercetin-3-glucuronide, M: myricetin, N: naringenin and EA: ellagic acid). In order to select the most active ones for further study, the authors of the present work studied 105 clinical isolates of 11 different strains of the following microbial species for the screening: *S. aureus*, *E. faecalis*, *Enterococcus faecium*, *E. coli*, *K. pneumoniae*, *Enterobacter* spp., *Serratia marcescens*, *Salmonella* spp., *P. aeruginosa*, *A. baumannii* and *Stenotrophomonas maltophilia.* The results showed that three pure compounds and two extracts were active against *S. aureus* and *S. maltophilia.* Due to the shortage of *S. maltophilia* isolates, they focused on *S. aureus*. In a second step, the most active compounds, and *C. salviifolius* and *P. granatum* extracts, were tested against the most sensitive species, those being 100 *S. aureus* (50 MRSA) clinical isolates. The best results with *S. aureus* strains were observed with the compound punicalagin and the extracts from *C. salviifolius* and *P. granatum*, with punicalagin being the main component of both extracts. The polyphenolic composition of the extracts is described in Table 2. The two polyphenolic extracts evaluated demonstrated different antibacterial properties depending on the antibiotic resistance profile of the bacteria tested, and this was related with their different polyphenolic composition: *C. salviifolius* extract, which contained hydrolysable tannins and flavonoids such as myricetin and quercetin derivates, was more effective against MRSA isolates, while *P. granatum* extract, containing mostly hydrolysable tannins, such as punicalin and punicalagin, demonstrated higher activity against methicillin-sensitive *S. aureus* (MSSA) isolates. The most abundant compounds in both extracts were hydrolysable tannins, so the differences in activity could be based on the minority compounds: the *P. granatum* extract was only composed of hydrolysable tannins (ellagitannins and gallotannins) and one flavonol at very low concentration, but the *C. salviifolius* extract contained more flavonoids (flavones, flavonols and flavanols), phenolic acids and a coumarin [33].

Another study demonstrated that the alcoholic extract from Thai mango seed kernel extract (MSKE) and its isolated phenolic principles not only were active against 19 clinical isolates of MRSA but also showed synergy with penicillin. Three polyphenols were found in MSKE: pentagalloylglucopyranose (PGG), methyl gallate (MG) and gallic acid (GA), with PGG being the most abundant and the one which showed the best antibacterial properties. Synergy in combination with penicillin G was also tested, and the results showed that MSKE and its phenolic principles increased the antibacterial activity of penicillin G against all tested isolates due to a bacteriostatic but not bactericidal effect. Although MG and GA alone exhibited a weak antibacterial effect against MRSA, results show their ability to enhance the bacterial susceptibility to penicillin G similarly to the alkyl gallates [34].

C. Bisignano et al. evaluated the antibacterial activity of polyphenol-rich fractions from raw shelled (NP) and roasted salted (RP) pistachios (*Pistacia vera* L.). Although they tested the extract against several Gram-negative and Gram-positive bacteria, yeasts and the fungus *Aspergillus niger*, only the *S. aureus* strains are clinical isolates, and hence subjects of this review. Forty-four clinical isolates of *S. aureus* (9 MRSA) from skin infections and surgical infections samples were tested. The major polyphenols detected in the pistachio samples were: hydroxybenzoic acids (as gallic and protocatechuic acids), flavan-3-ols (as (+)-catechin) and flavonols (as quercetin-3-*O*-glucoside). NP showed slightly higher total amounts of polyphenols, catechin and epicatechin, doubling their concentrations in NP. However, hydroxybenzoic acids and chlorogenic acid were more abundant in the roasted pistachios. Not only did both pistachio extracts present antibacterial activity against Gram-positive bacteria, but they also showed bactericidal effect against *S. aureus* (including MRSA) clinical isolates. Extracts from raw shelled pistachios were more active than those from roasted salted pistachios. A higher number of polyphenols determined in NP and the different qualitative composition of the two extracts could explain this behavior [35]. A few years later, the authors demonstrated that extracts from pistachios were also partially active against clinical strains of *Staphylococcus* spp. (some being multi-drug resistant). In this study 31 *Staphylococcus* clinical isolates were evaluated: 23 *S. aureus* (21 MRSA), 2 *S. epidermidis*, 2 *S. lugdunensis*, 2 *S. hominis*, 1 *S. xylosus* and 1 unidentified *Staphylococcus.* All strains were collected from orthopedic infections. The quantitative polyphenolic composition of pistachio extracts is described in Table 2. The total phenolic content was expressed as gallic acid equivalent (GAE)/100 g fresh weight (FW), with this value being higher in natural pistachios than in roasted ones, as described in previous research. According to the previous study, both NP and RP polyphenolic extracts were active against Gram-positive bacteria. However, the activity was bacteriostatic rather than bactericidal [36].

Other authors evaluated the ethanol crude extracts of 19 Chinese medicinal plants against 9 MRSA clinical strains. The article does not mention anything about the composition of the plants, except for *D. capitata*, which possesses polyphenols. The authors concluded that all the studied plant extracts showed antibacterial activity against the strains tested, with *D. capitata*, *Elsholtzia rugulosa*, *Elsholtzia blanda*, *Geranium strictipes* and *Polygonum multiflorum* being the ones with the best antibacterial properties. The antibacterial activity of one of the most active extracts from *D. capitata* and its fractions was further evaluated. The extract was suspended in water, and this suspension was fractionated between water and petroleum ether, chloroform, and ethyl acetate to form four fractions. All the fractions, except petroleum ether, showed antibacterial activity against MRSA. Only betulinic acid (the main compound present in the chloroform fraction) showed antibacterial activity against MRSA [37].

#### 2.2.2. *Helicobacter pylori*

In the Brown J.C. et al. study, several parts of different grape extracts (*Vitis vinifera* and *Vitis rotundifolia*) were tested: red grape skin, white grape skin, black grape skin, muscadine grape skin, muscadine grape seed and combinations of extracts from both plants. The major phenolic compounds of these plants were ellagic acid, myricetin, quercetin, and resveratrol. The extracts and the same pure polyphenols were studied against clinical strains of *Helicobacter pylori*, another high priority pathogen. These findings show significant but differing effects against *H. pylori* growth: muscadine grape skin extract was the most effective, followed by muscadine seed and combined extracts (skin and seed). Resveratrol and ellagic acid also inhibited *H. pylori;* however, myricetin had no effect. Although specific polyphenols of each individual extract are not defined in this study, the authors conclude that muscadine grape seed extract contained the highest total phenolic content, followed by muscadine combined with skin extracts. The major phenolics were ellagic acid, myricetin, quercetin and resveratrol. Another important conclusion suggested by the authors, in contrast with other experiments, is that higher phenolic levels were not always correlated with increased anti-*H. pylori* activity. The type and concentrations of the compounds present in these extracts played a more important role. Although anti-*H. pylori* activities by individual compounds were reported, it is believed that a synergistic mode of action is more likely responsible for the extract’s antibacterial activity. However, these complex interactions among compounds found in these extracts have yet to be determined [38].

### 2.3. Bacteria out of the Who Classification

Out of the WHO classification, there are also a vast number of pathogenic bacteria important in human health. We have found literature about the antibacterial activity of polyphenol-rich extracts against *S. maltophilia*, a relevant pathogenic Gram-negative bacterium which mostly causes infections in hospitalized patients with comorbidities and possesses intrinsic resistance to a high number of antibiotics. Theaflavin and its combination with epicatechin (2:1) (which showed better activity) [21] and EGCG [39] gave positive results against this resistant pathogen. In the former study, sputum samples were employed, whereas in the latter one, the authors used samples collected from the respiratory tract, bloodstream infections, catheter tips, wound drain fluids and ileal biopsy.

We also found information about two other Gram-positive pathogens: *Bacillus* sp. and *E. faecalis.* *C. longa* L. (Zingiberaceae) extracts showed antibacterial activity against clinical strains of *Bacillus* sp. [25] and *L. inermis* extracts [29], and extracts obtained from leaves and inflorescences of the vegetative organs of different species of *A. retroflexus* [23] showed antibacterial activity against *B. subtilis*. In the latter study, the *A. retroflexus* alcoholic extract showed the best activity against the *B. subtilis* strain among all microorganisms tested. Furthermore, the extracts were also assayed in combination with antibiotics to determine synergy, obtaining good results. The alcoholic extract of *A. retroflexus* leaves potentiated the antibacterial activity of trimetoprim-sulphamethoxazole, piperacillin-tazobactam, penicillin, cefaclor, chloramphenicol, kanamycin, and vancomycin against *B. subtilis.* The extract from the inflorescences of *A. retroflexus* improved the activity of cefaclor, chloramphenicol, kanamycin, and vancomycin against this Gram-positive bacterium. The best synergistic effect was observed with chloramphenicol against one of the of *B. subtilis* strains [23].

The previously described study by E. Sánchez et al. reports the antibacterial effect of *P. laevigata*, *O. ficus-indica*, *M. vulgare* and *S. drummondii* extracts on clinical isolates of *E. faecalis* [28].

**Table 2:** Phenolic-rich extracts which have demonstrated antibacterial activity against clinical isolates.

Botanical Extract	Phenolic Compounds	Microorganisms	Clinical Samples	Synergism with Antibiotics	Ref.
***S. baicalensis*** ***R. rugosa*** ***T. chebula***	Norwogonin, ellagic acid chebulagic acid, chebulinic acid, corilagin and terchebulin	*A. baumannii* (3 MDR strains)	1 blood, 2 respiratory	Ampicillin/sulbactam, azithromycin, cefepime, colistin, imipenem, levofloxacin, minocycline, rifampin, tobramycin, trimethoprim/sulfamethoxazole	[20]
**-**	**Epicatechin, theaflavin**	*A. baumannii* *S. maltophilia*	Sputum		[21]
***P. cuspidatum***		*S. aureus* (3 strains) *A. baumannii * (3 strains) *P. aeruginosa* (4 strains)	blood and sputum	Erytromycin, gentamicin, tetracycline, spectinomycin, piperacillin, G kanamycin, amikacin, clindamycin, ampicillin, cephalosporinin, trimethoprim/sulfamethoxazole, amoxillin	[22]
***A. retroflexus***	Not determined (literature: rich in polyphenols) *	*S. aureus * *B. subtilis * *P. aeruginosa* *E. coli* *K. pneumoniae* *A. baumannii*		Trimetoprim-sulphamethoxazole, piperacillin-tazobactam, ticarcillin-clavulanic acid, piperacillin, ceftriaxone, cefotaxime, cephalexin, penicillin, cefaclor, chloramphenicol, ciprofloxacin, kanamycin, colistin, vancomycin	[23]
	**Epigallocatechin gallate**	*P. aeruginosa* (15 MDR strains)		Aztreonam, cefotaxime	[24]
***C. longa* L. ** (Zingiberaceae)	Tannins, flavonoids	*E. coli* *P. aeruginosa* *K. pneumoniae* *Proteus* sp. *Salmonella* sp. *Bacillus* sp.			[25]
***A. nilotica*** ***C. zeylanicum*** ***S. aromaticum***	Not defined	*K. pneumoniae* *E. coli*			[26]
***S. secundiflora*** ***S. ambigua*** ***P. laevigata*** ***O. ficus-indica*** ***M. vulgare*** ***S. drummondi*** ***N. bivalve*** ***G. microcephala***	Tannins, flavonoids	*K. pneumoniae* *E. faecalis* *E. coli* *S. maltophilia* *S. aureus*			[28]
***L. inermis***	Tannins and other polyphenols	*E. coli * *S. typhi* *Klebsiella* spp. *S. sonnei * *B. subtilis * *S. aureus * *S. epidermidis*			[29]
***P. granatum.***	Pomegranate juice: anthocyanins (delphinidin, cyanidin glucosides), hydrolysable tannins (gallotannins), ellagitannins and gallagyl esters, hydroxybenzoic and hydroxycinnamic acids. Promegranate peel: bis-HHDP-hexoside (pedunculagin I), punicalin, ellagic acid (hexose, pentose and deoxyhexose derivatives)	*S.aureus* *E.coli*	Pharyngeal sample, sputum		[30]
***T. alternifolia***	Flavonoids	*S. aureus* (11 MRSA strains, 4 VRSA strains)	Skin infections		[31]
-	*Flavonols:* **morin, quercetin, kaempferol** *Flavanols and derivatives:* **(−)-epigallocatechin gallate, (+)-catechin acyl derivates, epicatechin gallate 3-*O*-decyl-(+)-catechin, (+)-catechin** *Phenolic acids and derivatives:* **protocatechuic acid, ethyl ester, caffeic acid**	*S. aureus*			[32]
***C. salviifolius*** ***C. paradisi*** ***P. granatum*** ***C. salviifolius*** ***C. paradisi*** ***P. granatum***	*C. salviifolius:* (epi)gallocatechin and its dimer pedunculagin I coumaroylquinic acid prodelphinidin B2-3′-*O*-gallate, punicalagin I and II, caffeoyl-hexose, myricetin hexoside, tergallic-C-glucoside, myricetin 3-arabinoside I and II, quercetin glucoside, ellagic acid-7-xiloside I and II, kaempferol diglycoside *P. granatum:* HHDP glucoside isomers, galloyl glucosa, punicalin, pedunculagin I, punicalagin isomers I, II and III, punigluconin, quercetin glucoside, ellagic acid rhamnoside, ellagic acid * gallic acid punicalagin, quercetin-3-glucuronide, myricetin, naringenin, **ellagic acid**	*S. aureus* (100 strains, 50 MRSA) *S. aureus* (100 strains, 50 MRSA)			[33]
***M. indica* L.**	Pentagalloylglucopyranose, methyl gallate and gallic acid **The same isolated phenolic principles**	*S. aureus* (19 strains MRSA)		Penicillin	[34]
***P. vera* L.**	Hydroxybenzoic acids (gallic acid, protocatechuic acid), flavan-3-ols (+-catechin), flavonols (isoquercetin) *	*S. aureus* (44 strains, 9 MRSA)	Skin and surgical infections		[35]
***P. vera* L.**	Hydroxybenzoic acids: gallic acid, protocathecuic acid, hydroxybenzoic acid, vanillic acid Hydroxycinnamic acids: chlorogenic acid, caffeic acid, cumaric acid Flavanones: eryodictiol, eryodictiol-7-*O*-glucoside, naringenin, naringin Flavonols: kaempferol-3-O-rutinoside, quercetin, quercetin-3-*O*-rutinoside, quercetin-3-*O*-glucoside Flavones: amentoflavone, luteolin, apigenin Isoflavones: daidzein, genistein Flavanols: epicatechin, catechin *	*Staphylococcus:* (31 strains) *S. aureus* (23, 21 MRSA) *S. epidermidis* (2) *S. lugdunensis* (2) *S. hominis* (1) *S. xylosus* (1) *Staphylococcus* (1 not identified)	Orthopedic infections: 16 knee prosthesis or surgical wounds, 7 hip prosthesis, and other orthopedic sites		[36]
19 Chinese medicinal plants: ***D. capitata***	Polyphenols	*S. aureus* (9 MRSA strains)			[37]
***V. vinifera*** ***V. rotundifolia***	**Ellagic acid, myricetin,** **quercetin, resveratrol**	*H. pylori*			[38]
**-**	**Epigallocatechin-3-gallate**	*S. maltophilia* (40 strains)	19 respiratory samples, 8 bloodstream infections, 7 catheter tips, 3 wounds, 2 drain fluids and 1 ileal biopsy		[39]

Polyphenols highlighted in bold are pure standards of synthetic origin. * Quantitative analysis of polyphenols is shown in the corresponding article.

**Table 3:** Phenolic-rich extracts which have demonstrated antifungal activity against fungal clinical isolates.

Botanical Extract	Phenolic Compounds	Microorganisms	Ref.
***A. retroflexus***	Not determined (literature: rich in polyphenols) *	*C. famata*, *C. utilis*, *C. albicans*, *S. cerevisiae*	[23]
***A. nilotica*, ** ***C. zeylanicum*** ***S. aromaticum***	Not defined	*C. albicans*	[26]
***D. longan***	Gallic acid, ellagic acid, corilagin * Reference compounds of the same polyphenols	*Candida species*, *C. neoformans* and some dermatophytes, *C. albicans* (9 strains), *C.neoformans* (4 strains), filamentous fungi (*T. rubrum*, *T. mentagrophytes*, *M. gypseum*, *M. canis*, *E. floccosum*, *P. boydii*, *P. siamensis*, *P. pinophilum*, *P. marneffei*, *A. nidulans*, *A. niger* and *A. fumigatus*)	[40]
	**Resveratrol**	*C. albicans* (30 strains) Fluconazole resistant strains of C. albicans ** (3 strains)	[41]

Polyphenols highlighted in bold are pure standards of synthetic origin. * Quantitative analysis of polyphenols is shown in the corresponding article. ** Synergism with azoles.

## 3. Antifungal Activity on Clinical Isolates

We have described all the literature regarding antibacterial activity of isolated polyphenols and polyphenol-rich extracts against a wide range of bacteria, including Gram-positive and Gram-negative bacteria. Some previous studies have demonstrated the antifungal activity of polyphenols and extracts as well, mostly against yeasts. *A.* *nilotica*, *C. zeylanicum* and *S. aromaticum* extracts showed antifungal properties against *C. albicans* [26]. Extracts obtained from leaves and inflorescences of the vegetative organs of different species of *A. retroflexus* were also active against *Candida famata*, *Candida utilis*, *C. albicans* and *Saccharomyces cerevisiae*, showing a major effect against *C. famata* [23].

Gallic acid, ellagic acid and corilagin were active against *Candida* species and *Cryptococcus neoformans*. Ellagic acid and longan (*Dimocarpus longan Lour*) seed extracts were active against *C. albicans* and *C. neoformans.* Corilagin and ellagic acid demonstrated a weak antifungal activity against *Trichophyton rubrum*, *Microsporum gypseum* and *Epidermophyton floccosum*, while Longan seed extract exhibited a weak antifungal effect on the tested species of dermatophytes [40].

One study used different longan extracts in order to investigate their antifungal activity against Candida species, C. neoformans and some fungi (*T. rubrum*, *Trichophyton mentagrophytes*, *M. gypseum*, *Microsporum canis*, *E. floccosum*, *Pseudallescheria boydii*, *Penicillium siamensis*, *Penicillium pinophilum*, *Penicillium marneffei*, *Aspergillus nidulans*, *A. niger* and *Aspergillus fumigatus*). The authors chose Longan cultivar Edor and Baidam extracts. Apart from these extracts, the following reference compounds were also used: gallic acid, ellagic acid, and corilagin. Dried seed contained the highest levels of these three compounds, followed by dried peel and dried pulp. Water extracts of longan fruit contained high levels of polyphenolic compounds such as corilagin, gallic acid, and ellagic acid, showing differences depending on the part of the plant; gallic acid, ellagic acid and corilagin are the predominant polyphenolic compounds of the dried longan seed extracts, while longan pulp extract showed a small amount of ellagic acid but no gallic acid and corilagin. With respect to the microbial activity due to the reference compounds, the following results were reported:

- Ellagic acid exhibited the best antifungal activity, followed by corilagin and gallic acid, respectively.
- The three polyphenolic compounds (gallic acid, ellagic acid, and corilagin) were active against *Candida* species and *C. neoformans*;
- Ellagic acid and longan seed extracts inhibited the growth of *C. albicans* and *C. neoformans* (with longan seed extract being more effective than pure ellagic acid). Ellagic acid showed better activity against *Candida parapsilosis* and *C. neoformans* than against *Candida krusei* and some *C. albicans* clinical strains;
- Gallic acid exhibited better antifungal activity against *C. parapsilosis* and most *C. albicans* strains than against *C. krusei* and *C. neoformans*;
- Corilagin demonstrated a low effect against both *Candida* sp. and *C. neoformans*;
- Regarding dermatophytes, ellagic acid demonstrated weak antifungal activity against *T. rubrum*, *M. gypseum* and *E. floccosum*. Gallic acid and corilagin could not inhibit the growth of these dermatophytes.

The results with longan extracts showed that:

- Longan seed extract demonstrated the best antifungal activity, while pulp and whole fruit extracts showed no effects;
- Longan seed extract (Baidam) was more active against *C. parapsilosis* than against *C. albicans*, *C. neoformans* and *C. krusei*, while longan seed extract (Edor) showed worse antifungal activity against *C. parapsilosis*, *C. krusei* and *C. neoformans* than Baidam. Edor did not show better antifungal activity than Baidam against *C. albicans.* Higher amounts of gallic acid and ellagic acid in Baidam seed extract could be related to its higher inhibitory activity on *C. krusei*, *C. parapsilosis* and *C. neoformans* than Edor seed extract;
- Regarding dermatophytes, longan seed extract exhibited a weak antifungal effect on the tested species of dermatophytes, including *T. rubrum*, *M. gypseum* and *E. floccosum*;
- None of the tested longan extracts showed antifungal activity against the filamentous fungi tested: *A. niger*, *A. nidulans*, *A. fumigatus*, *P. marneffei*, *P. siamensis*, *P. pinophilum*, *T. mentagrophytes*, *T. rubrum*, *M. gypseum*, *M. canis*, and *P.* *boydii* [40].

In the last study, it was demonstrated that trans-resveratrol (RSV) possessed a synergistic activity with azoles on the tested strains of *C. albicans* but showed no antifungal activity when used alone. Myxobacteria from soil samples were used. The authors determined the MIC50 values for ketoconazole, itraconazole and fluconazole against 30 clinical isolates of *C. albicans*, and synergy was demonstrated on more than 83% of the clinical strains tested using the combination of azoles and RSV. The authors found that the synergy varied when they used different culture media. RSV demonstrated its effectiveness against azole-resistant isolates as well. RSV could also significantly enhance the antifungal susceptibility of two of the three resistant fluconazole *C. albicans* strains tested. Itraconazole and ketoconazole in combination with RVS were then used against the resistant strain, obtaining antifungal effects; this indicates that when RSV alone is not effective, a combination with other azole drugs is an alternative strategy [41].

## 4. Concluding Remarks

Antibiotic resistance is a global issue which affects a great number of areas and makes it urgent to find new ways to fight against resistant bacteria. Polyphenols and polyphenolic-rich extracts could help to overcome this problem, since many studies have reported their antimicrobial activity against some pathogenic microbial species. Unfortunately, there is little research studying their effectiveness on clinical isolates. In this review we have gathered all the information related to this topic, showing that polyphenols could be a promising key to combat resistant bacteria and reduce drug dosage with all the benefits that this practice may entail. Many studies show how these compounds exhibit antimicrobial activity, either when using isolated molecules or polyphenolic-rich plant extracts. In this latter case, it has been reported that higher polyphenolic levels do not always mean a better antimicrobial activity, but this is mostly due to the qualitative composition of the extract and the relative concentration of each compound [38]. Some of the studies reviewed showed that polyphenols act synergistically in combination with antibiotics.

Although in vitro experiments seem promising, further studies are needed to prove the usefulness of polyphenols in the treatment and prevention of human infectious diseases. The use of polyphenolic-based disinfection products could prevent the emergence of such diseases in a friendly way. The development of topical pharmaceutical formulations such as creams, ointments or lotions containing polyphenols as enhancers of the activity of clinically used antibiotics, could also be a way to reduce the occurrence of resistance to these indispensable drugs. Even though the way to the actual use of the proposed alternative to antibiotics passes through the development of the aforementioned formulations and the implementation of clinical trials to assess effectiveness, these are steps worth taking if we consider the evolution of resistance in recent years and the uncertain future of the present treatments.

## Figures and Tables

**Table 1:** Classification of the main active polyphenols against clinical isolates reviewed.

Number and Layout of Carbon Atoms	Polyphenol Class or Family (*Polyphenol subclass*)	Reviewed Polyphenols
**C_6_–C_1_**	**Benzoic acids**	Gallic acid
		Methyl gallate
		Protocathecuic acid
		Protocatechuic acid ethyl ester
		Hydroxybenzoic acid
		Vanillic acid
**C_6_–C_3_**	**Cinnamic acids**	Chlorogenic acid
		Caffeic acid
		Coumaric acid
		Coumaroylquinic acid
		Caffeoyl-hexose
	**Coumarins**	
**C_6_–C_2_–C_6_**	**Stilbenes**	Resveratrol
		Piceid
**C_6_–C_3_–C_6_**	**Flavonoids**	
	*Flavonols*	Quercetin
		Quercetin-3-*O*-rutinoside
		Quercetin-3-*O*-glucoside
		Myricetin
		Kaempferol
		Kaempferol diglycoside
		Kaempferol-3-*O*-rutinoside
		Morin
	*Flavones*	Apigenin
		Luteolin
		Amentoflavone
		Norwogonin
	*Flavan-3-ols*	Catechin
		Epicatechin
		Epicatechin gallate
		Epigallocatechin-3-gallate
		3-*O*-decyl-(+)-catechin
		Catechin acyl derivates
		Prodelphinidin B2-3′-*O*-gallate
		Theaflavin
	**Flavonoids *(cont.)***	
	*Flavanones*	Naringenin
		Naringin
		Eryodictiol
		Eryodictiol-7-*O*-glucoside
	*Isoflavones*	Daidzein
		Genistein
	*Anthocyanidins*	Delphinidin glucosides
		Cyaniding glucosides
**(C_6_–C_1_)_n_**	**Hydrolysable tannins**	
	*Building blocks*	Ellagic acid
		Pentagalloylglucopyranose
		Hexahydroxydiphenic acid (HHDP)
	*Ellagitannins*	Corilagin
		Chebulinic acid
		Chebulagic acid
		Terchebulin
		Punicalin
		Pedunculagin I
		Punicalagin I, II and III
		Punigluconin
		Dehydrated tergallic-c-glucoside
		HHDP glucoside isomers
	*Gallotannins*	Galloyl glucose
	*Gallagyl esters*	

## References

[1] Agencia Española de Medicamentos y Productos Sanitarios (AEMPS) Nota Informativa 11/2010 Los Líderes Mundiales Reunidos en la Asamblea General de las Naciones Unidas se Comprometen a Adoptar una Estrategia Contra la Resistencia a los Antibióticos. https://www.aemps.gob.es/informa/notasInformativas/laAEMPS/2016/docs/NI-AEMPS_11-2016-reunion-ONU-antibioticos.pdf?x89163.

[2] AEMPS Plan Nacional Frente a la Resistencia a los Antibióticos 2019–2021. http://www.resistenciaantibioticos.es/es/system/files/field/files/pran_2019-2021_0.pdf?file=1&type=node&id=497&force=0.

[3] Écoantibio 2: Plan National de Réduction des Risques D’antibiorésistance en Médecine Vétérinaire (2017–2022). https://agriculture.gouv.fr/le-plan-ecoantibio-2-2017-2022..

[4] DART 2020 Fighting Antibiotic Resistance for the Good of both Humans and Animals. https://www.bmel.de/SharedDocs/Downloads/EN/Publications/DART2020.pdf;jsessionid=C332D8B25955F30CC57A8BDFF94EB91D.live852?__blob=publicationFile&v=3.

[5] Swiss Antibiotic Resistance Report 2020. https://www.star.admin.ch/star/en/home.html.

[6] WHO Publishes List of Bacteria for Which New Antibiotics Are Urgently Needed. https://www.who.int/news/item/27-02-2017-who-publishes-list-of-bacteria-for-which-new-antibiotics-are-urgently-needed.

[7] Asokan G.V., Ramadhan T., Ahmed E., Sanad H. (2019). WHO global priority pathogens list: A bibliometric analysis of medline-pubmed for knowledge mobilization to infection prevention and control practices in Bahrain. Oman Med. J..

[8] Rice L.B. (2008). Federal funding for the study of antimicrobial resistance in nosocomial pathogens: No ESKAPE. J. Infect. Dis..

[9] Mulani M.S., Kamble E.E., Kumkar S.N., Tawre M.S., Pardesi K.R. (2019). Emerging strategies to combat ESKAPE pathogens in the era of antimicrobial resistance: A review. Front. Microbiol..

[10] Daglia M. (2012). Polyphenols as antimicrobial agents. Curr. Opin. Biotechnol..

[11] Fraga C.G., Croft K.D., Kennedy D.O., Tomás-Barberán F.A. (2019). The effects of polyphenols and other bioactives on human health. Food Funct..

[12] Efenberger-Szmechtyk M., Nowak A., Czyzowska A. (2021). Plant extracts rich in polyphenols: Antibacterial agents and natural preservatives for meat and meat products. Crit. Rev. Food Sci. Nutr..

[13] Olszewska M.A., Gędas A., Simões M. (2020). Antimicrobial polyphenol-rich extracts: Applications and limitations in the food industry. Food Res. Int..

[14] Skroza D., Šimat V., Smole Možina S., Katalinić V., Boban N., Generalić Mekinić I. (2019). Interactions of resveratrol with other phenolics and activity against food-borne pathogens. Food Sci. Nutr..

[15] Bouarab Chibane L., Degraeve P., Ferhout H., Bouajila J., Oulahal N. (2019). Plant antimicrobial polyphenols as potential natural food preservatives. J. Sci. Food Agric..

[16] Gutiérrez-del-Río I., Fernández J., Lombó F. (2018). Plant nutraceuticals as antimicrobial agents in food preservation: Terpenoids, polyphenols and thiols. Int. J. Antimicrob. Agents.

[17] Rama J.L.R., Mallo N., Biddau M., Fernandes F., de Miguel T., Sheiner L., Choupina A., Lores M. (2021). Exploring the powerful phytoarsenal of white grape marc against bacteria and parasites causing significant diseases. Environ. Sci. Pollut. Res. Int..

[18] Álvarez-Martínez F.J., Barrajón-Catalán E., Encinar J.A., Rodríguez-Díaz J.C., Micol V. (2020). Antimicrobial Capacity of Plant Polyphenols against Gram-positive Bacteria: A Comprehensive Review. Curr. Med. Chem..

[19] Gato E., Rosalowska A., Martínez-Guitián M., Lores M., Bou G., Pérez A. (2020). Anti-adhesive activity of a *Vaccinium corymbosum* polyphenolic extract targeting intestinal colonization by Klebsiella pneumoniae. Biomed. Pharmacother..

[20] Miyasaki Y., Rabenstein J.D., Rhea J., Crouch M.L., Mocek U.M., Kittell P.E., Morgan M.A., Nichols W.S., van Benschoten M.M., Hardy W.D. (2013). Isolation and Characterization of Antimicrobial Compounds in Plant Extracts against Multidrug-Resistant *Acinetobacter baumannii*. PLoS ONE.

[21] Betts J.W., Kelly S.M., Haswell S.J. (2011). Antibacterial effects of theaflavin and synergy with epicatechin against clinical isolates of *Acinetobacter baumannii* and *Stenotrophomonas maltophilia*. Int. J. Antimicrob. Agents.

[22] Su P.W., Yang C.H., Yang J.F., Su P.Y., Chuang L.Y. (2015). Antibacterial activities and antibacterial mechanism of polygonum cuspidatum extracts against nosocomial drug-resistant pathogens. Molecules.

[23] Marinaş I.C., Chifiriuc C., Oprea E., Lazăr V. (2014). Antimicrobial and antioxidant activities of alcoholic extracts obtained from vegetative organs of *A. retroflexus*. Roum. Arch. Microbiol. Immunol..

[24] Betts J.W., Hornsey M., Higgins P.G., Lucassen K., Wille J., Salguero F.J., Seifert H., la Ragione R.M. (2019). Restoring the activity of the antibiotic aztreonam using the polyphenol epigallocatechin gallate (EGCG) against multidrug-resistant clinical isolates of Pseudomonas aeruginosa. J. Med. Microbiol..

[25] Álvarez N.M., Ortíz A.A., Martínez O.C. (2016). In Vitro antibacterial activity of Curcuma longa (Zingiberaceae) against nosocomial bacteria in Montería, Colombia. Rev. Biol. Trop..

[26] Khan R., Islam B., Akram M., Shakil S., Ahmad A., Ali S.M., Siddiqui M., Khan A.U. (2009). Antimicrobial activity of five herbal extracts against Multi Drug Resistant (MDR) strains of bacteria and fungus of clinical origin. Molecules.

[27] Zhang N., Liu W., Qian K. (2020). In-Vitro antibacterial effect of tea polyphenols combined with common antibiotics on multidrug-resistant Klebsiella pneumoniae. Minerva Med..

[28] Sánchez E., Rivas Morales C., Castillo S., Leos-Rivas C., García-Becerra L., Ortiz Martínez D.M. (2016). Antibacterial and Antibiofilm Activity of Methanolic Plant Extracts against Nosocomial Microorganisms. Evid. Based Complement. Alternat. Med..

[29] Gull I., Sohail M., Aslam M.S., Athar M.A. (2013). Phytochemical, toxicological and antimicrobial evaluation of lawsonia inermis extracts against clinical isolates of pathogenic bacteria. Ann. Clin. Microbiol. Antimicrob..

[30] Pagliarulo C., de Vito V., Picariello G., Colicchio R., Pastore G., Salvatore P., Volpe M.G. (2016). Inhibitory effect of pomegranate (*Punica granatum* L.) polyphenol extracts on the bacterial growth and survival of clinical isolates of pathogenic *Staphylococcus aureus* and *Escherichia coli*. Food Chem..

[31] Marathe N.P., Rasane M.H., Kumar H., Patwardhan A.A., Shouche Y.S., Diwanay S.S. (2013). In Vitro antibacterial activity of *Tabernaemontana alternifolia* (Roxb) stem bark aqueous extracts against clinical isolates of methicillin resistant Staphylococcus aureus. Ann. Clin. Microbiol. Antimicrob..

[32] Miklasińska-Majdanik M., Kępa M., Wojtyczka R.D., Idzik D., Wąsik T.J. (2018). Phenolic Compounds Diminish Antibiotic Resistance of *Staphylococcus Aureus* Clinical Strains. Int. J. Environ. Res. Public Health.

[33] Álvarez-Martínez F.J., Rodríguez J.C., Borrás-Rocher F., Barrajón-Catalán E., Micol V. (2021). The antimicrobial capacity of *Cistus salviifolius* and *Punica granatum* plant extracts against clinical pathogens is related to their polyphenolic composition. Sci. Rep..

[34] Jiamboonsri P., Pithayanukul P., Bavovada R., Chomnawang M.T. (2011). The inhibitory potential of thai mango seed kernel extract against methicillin-resistant staphylococcus aureus. Molecules.

[35] Bisignano C., Filocamo A., Faulks R.M., Mandalari G. (2013). In Vitro antimicrobial activity of pistachio (*Pistacia vera* L.) polyphenols. FEMS Microbiol. Lett..

[36] La Camera E., Bisignano C., Crisafi G., Smeriglio A., Denaro M., Trombetta D., Mandalari G. (2018). Biochemical Characterization of Clinical Strains of *Staphylococcus* spp. and Their Sensitivity to Polyphenols-Rich Extracts from Pistachio (*Pistacia vera* L.). Pathogens.

[37] Zuo G.Y., Wang G.C., Zhao Y.B., Xu G.L., Hao X.Y., Han J., Zhao Q. (2008). Screening of Chinese medicinal plants for inhibition against clinical isolates of methicillin-resistant Staphylococcus aureus (MRSA). J. Ethnopharmacol..

[38] Brown J.C., Huang G., Haley-Zitlin V., Jiang X. (2009). Antibacterial effects of grape extracts on helicobacter pylori. Appl. Environ. Microbiol..

[39] Gordon N.C., Wareham D.W. (2010). Antimicrobial activity of the green tea polyphenol (-)-epigallocatechin-3-gallate (EGCG) against clinical isolates of *Stenotrophomonas maltophilia*. Int. J. Antimicrob. Agents.

[40] Rangkadilok N., Tongchusak S., Boonhok R., Chaiyaroj S.C., Junyaprasert V.B., Buajeeb W., Akanimanee J., Raksasuk T., Suddhasthira T., Satayavivad J. (2012). In Vitro antifungal activities of longan (*Dimocarpus longan* Lour.) seed extract. Fitoterapia.

[41] Wang J., Zhang X., Gao L., Wang L., Song F., Zhang L., Wan Y. (2021). The synergistic antifungal activity of resveratrol with azoles against Candida albicans. Lett. Appl. Microbiol..

